# The Effectiveness of EMOVERE: An Emotional Education Program for Young Couples

**DOI:** 10.3390/ijerph18041677

**Published:** 2021-02-09

**Authors:** Estefanía Mónaco, Usue de la Barrera, Inmaculada Montoya-Castilla

**Affiliations:** Department of Personality, Assessment and Psychological Treatments, Faculty of Psychology, University of Valencia, 46010 Valencia, Spain; estefania.monaco@uv.es (E.M.); usue.barrera@uv.es (U.d.l.B.)

**Keywords:** emotion regulation, program evaluation, intervention, couples, young adulthood, emotional competences, attachment

## Abstract

Our aim was to assess the effectiveness of EMOVERE, a psychoeducational and experiential program to increase emotion regulation in couples. Forty-four young couples (*n* = 88) aged between 18 and 36 years old participated in the study (53.4% women; *M* = 24.18; *SD* = 4.34). Twenty-two couples belonged to the experimental group (received the intervention) and 22 to the control group (received no intervention). The intervention program consisted of seven two-hour sessions over a month, in groups of four to five couples. The variables studied were sociodemographic characteristics, emotional intelligence (TMMS-24), emotional inter-regulation with the partner (SIERC), attachment (ECR-S) and satisfaction with the relationship (RAS). The proposed design was quasi-experimental, with two randomized groups (experimental and control group) and longitudinal data from two occasions. SPSS version 24.0 was used to perform analysis of variance (MANOVA and MANCOVA), multiple hierarchical regression and reliable change index. PROCESS was also used for moderation analyses. The results indicate that the program is effective in increasing emotional self-regulation and emotion regulation with the partner, as well as reducing couples’ avoidance of intimacy. Age, relationship duration and previous relationship satisfaction moderate the effectiveness of the program. The importance of continuing this research line to address well-being of young populations is discussed.

## 1. Introduction

### 1.1. Healthy Couple Relationships in Youth

A couple relationship can be defined as a relatively stable sentimental bond between two people who share emotional and intellectual intimacy, establish a commitment by planning shared projects and define a joint identity as a couple, making it visible to others [1]. In western societies, the couple is usually considered one of the highest priority social bonds, particularly during youth [2]. Youth is a vital period of change and adaptation to adult roles, when people invest a large proportion of their personal time and resources in establishing and maintaining a satisfactory couple relationship [3]. According to previous studies, people’s assessment of satisfaction with their sentimental relationship has a strong influence on their well-being [4], their quality of life [1], their happiness [5] and their overall health [6]. Hence, there is a social need to educate young people on how to establish healthy and violence-free relationships [7].

### 1.2. Emotional Competences to Improve Relationship Quality

One way to improve the quality of relationships is to acquire personal skills, such as emotional competences [8]. Emotional competences are defined as emotional skills that are developed in the immediate social context, in response to personal needs and the demands of the environment [9]. Well-developed emotional competences foster the ability to perceive and consider one’s partner’s feelings, as well as to understand, name and adequately express one’s emotional states [9]. All this leads to a better management of emotions, resolving relationship conflicts in a constructive way, without resorting to aggressiveness [10]. A high emotional competence therefore not only increases relationship satisfaction, but also prevents situations of violence in the relationship and increases the quality of the bond and the adjustment in the relationship [11,12].

Within the emotional competences, emotional self-regulation and inter-regulation seem to have an especially strong influence on couple relationships’ well-being and stability [13]. Emotional self-regulation refers to “those processes by which people influence the emotions we have, when we have them, and how we experience and express them” [14]. On the other hand, emotional inter-regulation is defined as the deliberate attempt to influence the emotions or moods of other people, concretely, in this case, the sentimental partner [15]. Both intra- and interpersonal emotion regulation are constantly set in motion in a couple’s daily life and affect its functioning [16]. These emotional skills can be learned and trained voluntarily, although they are also affected by other dispositional personal factors, such as attachment style [17]. Previous studies suggest that people with insecure attachment, both anxious and avoidant, have greater difficulties in understanding and regulating their own and their partners’ emotional states than people with a secure attachment style [18].

### 1.3. Why Do We Need Effective Interventions in Emotional Education for Couples?

Previous studies with dyads suggest that a person’s emotion regulation not only influences one’s own well-being and health, but also indirectly influences their partner’s [19]. In addition, learning emotional skills may reduce a major public health concern, intimate partner violence, even in people with a vulnerable predisposition, such as high levels of childhood trauma and insecure attachment [20]. These people tend to show emotional dysregulation, characterized by nonacceptance of emotional responses, difficulties engaging in goal-directed behaviour and impulse control, lack of emotional awareness and clarity and limited access to emotion regulation strategies [16]. This is an antecedent to aggression and violent behaviour [21]. On the other hand, active engagement, constructive problem solving, optimism, positive self-verbalization and reframing of the situation were among the more functional coping behaviors and were positively related to higher relationship quality [22].

In order to help couples to maintain healthy, mutually satisfying and stable relationships, relationship education programs have been developed. Relationship education programs are psychoeducational interventions designed to prevent future relationship problems by developing relationship skills and disseminating knowledge about life as a couple [23]. Education can be helpful in adjusting unhealthy expectations of relationships by reducing beliefs in romantic love myths, decreasing acceptance of violence in relationships, managing conflict and practicing communication skills. Additionally, some authors suggest that other skills that are less frequently worked on in relationship education programs, such as emotional regulation, may be critical to improving relationships [24].

The first relationship education program was developed, called The Couple Communication Program, which demonstrated moderate to large effects on relationship satisfaction at the end of the program and follow-up [25]. Following this pioneering work, more programs continued to be developed in subsequent years, and especially in the 1990s. There were six important programs developed in that decade, according to the strength of their rationale and their empirical evaluation (considering longitudinal data analysis, predictive and outcome research, comparison studies and meta-analyses, among others). They were as follows: Couples Communication (CC; [26]), The Marriage Survival Kit [27], PREPARE/ENRICH [28], Practical Application of Intimate Relationship Skills (PAIRS; [29]) and the Prevention and Relationship Enhancement Program (PREP; [30]), among others.

Relationship education programs have been shown to be effective in improving the quality of relationships and reducing partner distress [31,32,33]. The literature suggests that personal variables such as subjective well-being and happiness can be improved by intervening in the dyad [34]. Results suggest that effective partner interventions have positive consequences, such as reduced psychological abuse, increased positive communication and reduced conflict [35,36,37]. According to conducted meta-analysis studies, relationship education programs have small to moderate effects on partner satisfaction [38,39]. They are also effective in treating depression and substance use, as well as reducing pain symptoms and improving quality of life in patients with chronic diseases [40]. In addition, couple’s interventions have direct biological effects, such as reducing cortisol—the stress hormone—and systolic blood pressure [41,42]. The meta-analysis by Simpson, Leonhard and Hawkins [23] found significant mean effect sizes (studies with control group d = 0.36; studies with pre-post d = 0.47), including effects on attitudes and skills.

Following an extensive review of the most current literature, a number of relationship education programs developed in recent years have been found to have effective outcomes for couples’ well-being [31,32,33,37,43]. One of the most remarkable for us, because of its relationship with the theme of this research, is the “Hold Me Tight” program (HMT; [24]) Its goal is to help couples improve the process of regulating and sharing emotions and needs related to attachment in a way that promotes secure attachment between partners. Based on the strong association between secure attachment and relationship satisfaction, trust, intimacy and positive communication patterns, Kennedy et al. [24] suggest that the incorporation of attachment style work into relationship education programs can significantly increase their effectiveness.

Therefore, emotional education of the couple from youth is a social need that has to be covered through the implementation of validated and effective interventions [39]. Nevertheless, there are some methodological weaknesses in the demonstration of the effectiveness of a large part of the intervention programs applied in couples: evaluation with valid and reliable quantitative data, comparison of an experimental group with a control group or longitudinal evaluation of the effects, to give some examples [44]. Some of these interventions have been carried out with populations with specific characteristics, such as couples with a new-born [45], with fertility difficulties [46] or couples in which one of the members has a chronic disease [47]. Other programs have focused on couples with certain sociodemographic variables, such as low socioeconomic status or education [48], cultural background [49] or non-normative sexual orientation [43]. However, no studies have been found in young Spanish couples that promote health and well-being through education of emotional competences in sentimental relationships. Therefore, it is important to evaluate the effectiveness of dyadic interventions in emotional education in couples, based on a strong theoretical framework and with attention to the methodology used [50].

### 1.4. Theoretical Framework Behind EMOVERE Program 

To promote the effectiveness of interventions, as well as a careful methodology, it is imperative to base them on sound theoretical frameworks [44,51]. The EMOVERE program is based on the theory of vulnerability–stress–adaptation, as well as on the contrasted model of emotion regulation which in turn has been adapted to the emotional inter-regulation of couples.

The vulnerability–stress–adaptation model of relationship functioning [52] proposes that a couple’s ability to cope with external stressors predicts their relationship outcomes. Specifically, this model posits that three factors can interact and influence relationship satisfaction. First, each partner brings certain qualities to the relationship (e.g., communication style, family history) that render them more or less vulnerable to relationship challenges. Second, the context in which the relationship occurs may or may not have stressors that can impact relationship quality. Finally, the presence or lack of adaptive processes—such as cognitions, affect and behaviors that occur within each partner and the dyad—can also contribute to relationship satisfaction [43].

On the other hand, the emotion regulation model based on emotional processing [34] suggests that an active process of emotional acceptance, understanding and processing is necessary for an adequate emotion regulation. Hervás [53] suggests six tasks or processes that allow optimal emotional processing of the experience and, consequently, effective emotion regulation. These processes are: (1) emotional openness (capacity to consciously access body sensations and emotions); (2) emotional attention (dedicating attentional resources to emotional information); (3) emotional acceptance (absence of negative judgment in the face of emotional experiences); (4) emotional labelling (capacity to clearly name emotions); (5) emotional analysis (thinking about the meaning and different causes of emotions); and (6) emotional modulation (capacity to modulate emotional responses through emotional, cognitive or behavioural strategies). As will be commented on later, this model of intrapersonal emotion regulation was adapted for interpersonal emotion regulation in couple relationships.

### 1.5. Objective and Hypothesis of the Present Study

The objective of the present work was to develop and assess the effectiveness of the program EMOVERE. We propose three hypotheses: (1) the intervention will be effective in developing emotional competences (intrapersonal and interpersonal emotional skills) in partners; (2) the program will also reduce avoidance of intimacy and attachment anxiety associated with insecure attachment styles; and (3) the demographic variables (gender and age) as well as the relationship variables (relationship duration and relationship satisfaction) will moderate the effectiveness of the intervention.

## 2. Materials and Methods

### 2.1. Participants

**Figure 1:**
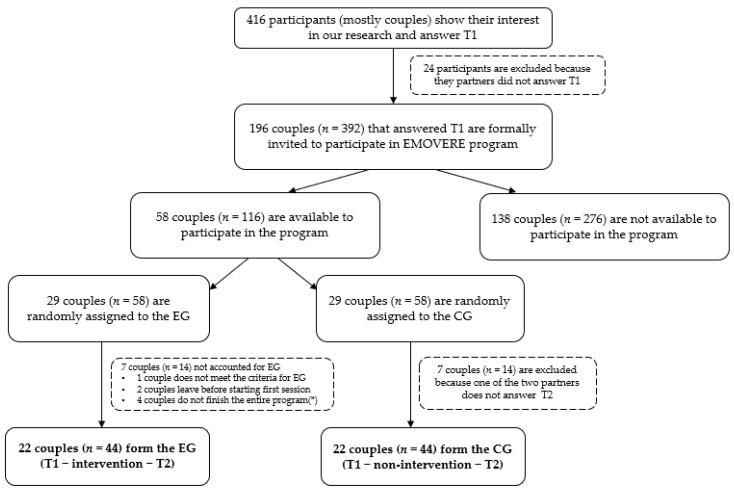
Sample chart. Note. T1 = pre-test; T2 = post-test; EG = experimental group; CG = control group; (*) one couple left for personal reasons, three couples for external reasons (COVID-19 pandemic).

The sample was collected through Internet promotions, and through posters and brochures in universities. As we can see in the sample chart (Figure 1), 416 people showed interest in our research and answered the first evaluation. The majority of people participated together with their partner, as requested. However, some of them answered individually without their partners, so they were not considered. The remaining 392 people were couples (192 couples in total). These couples were informed by the researchers about the intervention program EMOVERE and were invited to participate, confirming their interest and availability to participate in the program. Fifty-eight couples were available and met the criteria for our program. The inclusion criteria for participation in the study were to be between 18 and 36 years old and to have been in the relationship for more than 6 months. For participating in the experimental group, the requirements were that both members could attend all sessions in person, and that there were no previous serious relationship problems or psychological problems, since this is a psychoeducational program for prevention.

Half of the couples who met the criteria (29 couples) were randomly assigned to the experimental group, and the other half (29 couples) to the control group. Randomization was performed by a simple coin flip carried out by a person who was blind to the different conditions. The couples in the control group were registered on a waiting list to receive the program later. A brief pre-interview was conducted with the participants of the experimental group before starting the program, to confirm that they met the established criteria and know their expectations. Finally, 7 couples were excluded from the experimental group (1 couple did not meet the criteria of previous relationship problems and was recommended to attend specialized couple therapy [34]; 2 couples dropped out before the first session; 4 couples did not complete the program, 1 for personal reasons and 3 because the program was canceled due to the COVID-19 pandemic). In addition, 7 couples were excluded from the control group because one of the two members did not answer the post-test).

Consequently, the experimental group was formed of 22 couples (*n* = 44; 54.5% women; *M_age_* = 22.77 years; *SD* = 3.33) and the control group of 22 couples (*n* = 44; 52.3% women; *M_age_* = 25.59 years; *SD* = 4.80). None of the participating couples had dependent children. Regarding the sexual orientation of the participants in both groups, 77 (87.5%) were heterosexuals, 5 (5.7%) were homosexuals 5 (5.7%) were bisexuals and 1 (1.1%) was of another orientation. Of the participants, 27.3% lived with their partner, with a length of cohabitation ranging from 2 months to 10 years (M = 31.17 months; SD = 36.42). 

### 2.2. Instruments

Socio-demographic variables. Gender, age, sexual orientation, relationship duration and cohabitation with the partner were assessed by an ad hoc questionnaire.

Emotional intelligence. Emotional intelligence was evaluated through the Trait Meta-Mood Scale-24 (TMMS-24; developed by [54] and validated in Spanish population by [55]). The scale is composed of 24 items with a five-point Likert scale (1 = No agreement; 5 = Complete agreement) that evaluates three factors: (1) Attention: observation and thoughts about own emotions (e.g., “I usually spend time thinking about my emotions”; (2) Clarity: understanding of own emotional states (e.g., “I am clear about my feelings”); and (3) Repair: ability to regulate own emotions (e.g., “Although I sometimes feel sad, I usually have an optimistic view”). All three factors were highly reliable both in the Spanish validation (α = 0.88, α = 0.91 and α = 0.85, respectively) and in the present study (α = 0.87, α = 0.87 and α = 0.84).

Couple’s emotional inter-regulation. The Interpersonal Emotion Regulation for Couples Scale (SIERC; developed and validated in an original Spanish form by [56]) was used. It is composed of two forms (Form A and Form B) with 24 items each and answers from one to five (1 = Almost never; 5 = Almost always). Both forms of the questionnaire are answered by the same respondent. The respondent will be called “actor” from now on, and the respondent’s partner will be called “partner”. This instrument is composed of three dimensions (“Emotional expression”, “Emotional understanding” and “Emotional management”) with two different forms (Form A and Form B). Form A (SIERC-A) assesses the actor’s perception of their own abilities to inter-regulate the partner’s emotions, and Form B (SIERC-B) assesses the actor’s perception of the partner’s ability to inter-regulate emotions with the actor. Three dimensions are described as follows: (1) Emotional expression: in Form A, this dimension assesses the actor’s ability to express emotions in front of the partner (e.g., “I avoid being emotional in front of my partner”); in Form B, this dimension assesses the actor’s perception of the partner’s ability to express emotions in front of the actor (e.g., “My partner avoids being emotional in front of me”). (2) Emotional understanding: in Form A, this dimension measures the actor’s ability to attend to and understand the partner’s emotions (e.g., “I often ask my partner about how they are feeling”); Form B assesses the actor’s perception of the partner’s ability to attend and understand the actor’s emotions (e.g., “My partner often asks me how I am or how I am feeling”). (3) Emotional management: in Form A, this factor assesses the actor’s ability to validate and soothe the partner’s emotions (e.g., “My partner can freely express their emotions to me, because I am not judgmental about them”); Form B measures the actor’s perception of the partner’s ability to validate and soothe the actor’s emotions (e.g., “I can freely express my emotions to my partner, because they are not judgmental about them”). The reliability of the scale in our sample was good (Form A: α = 0.85; α = 0.81; and α = 0.78; Form B: α = 0.84; α = 0.86; and α = 0.85, respectively).

Adult attachment. The Experiences in Intimate Relationships questionnaire (ECR-S; developed by [57] and validated version in Spanish by [58]) was used to measure attachment. This instrument consists of 36 items with a seven-point Likert scale (1 = Totally disagree; 7 = Totally agree). It assesses two dimensions of attachment: anxiety (fear of rejection and abandonment by romantic partners) and avoidance (the degree to which the person feels uncomfortable maintaining emotional intimacy with others). The reliability of the scale in our sample was good (anxiety: α = 0.81; avoidance: α = 0.89).

Relationship satisfaction. Relationship satisfaction was assessed using the Relationship Rating Scale (RAS; developed by [59] and validated version in Spanish by [60]). This consists of seven items in a 5-point Likert scale (1 = Strongly Disagree; 5 = Strongly Agree). The questions refer to the degree of satisfaction, quality of the relationship, the coverage of needs and difficulties in the relationship. This instrument provides an overall score that indicates the degree of general relationship satisfaction, with reliability (α) of 0.86. The reliability for this study was good (α = 0.81).

### 2.3. Procedure

The couples in the experimental group were divided into subgroups of 4 or 5 couples each. The program was applied in an adapted university classroom outside of class hours. Participation was completely voluntary and without financial or other compensation. The intervention groups were held at different times between January 2019 and March 2020. The control group participants also responded to the proposed battery over this period.

The evaluation of the participants was longitudinal, on two occasions. In the experimental group, a pre-evaluation was carried out before the start of the program, and a post-evaluation one and a half months later, after the program had been completed. In the control group, a pre-evaluation was carried out, and a post-evaluation after one and a half months, without any intervention.

Both evaluations of the participants were carried out online through the Limesurvey platform. The indications were to fill in the battery of questionnaires individually, without being in the presence of the couple or commenting on the answers. To respect their anonymity, the couples were asked to associate themselves by means of the same non-identifiable code linked with their identity. The study was carried out following the guidelines of the Helsinki Declaration [61] and the Council of Europe Convention on Human Rights, and with the approval of the Ethics Committee of the University of Valencia (H152846236674). All participants completed the corresponding informed consent and were duly informed of the procedure as well as of the possibility of revoking their consent at any time.

### 2.4. Statistical Analyses

SPSS version 24.0 (IBM Corp., Armonk, NY, USA) [62] was used to carry out the data analysis. To study the impact of the intervention program, multivariate analyses of variance (MANOVA) were performed with pre-intervention scores in the experimental and control groups to identify possible differences at baseline. Multivariate covariance analyses (MANCOVA) were performed to identify changes at post-intervention, controlling for pre-intervention scores as covariables. In addition, the effect size (Cohen’s d) of each variable was calculated to estimate the magnitude of the differences between the experimental and control groups [63]. Multiple hierarchical regression analyses were also conducted to examine the effectiveness of the intervention program [64]. We calculated the within-person change in attachment and emotional skills from pre- to post-intervention by subtracting the values of T1 from those of T2 and introduced these values as the dependent variables. As independent variables, we introduced the T1 score (control variable) in the first step, and the experimental condition (1 = experimental group, 0 = control group) in the second step. Predictions based on the experimental condition, controlling for the pre-intervention score, are interpreted to mean that significant change is attributed to the intervention program. 

Later, the reliable change index (RCI) was calculated, which evaluates the reliable change by means of the variation of the standard error (SE) of the measurement that considers the two evaluations that have been carried out. Reliable change refers to the extent to which the change shown by an individual is outside the range that could be attributed to the variability inherent in measurement [65]. Chi-square tests were performed to compare the percentage of participants in both groups who had a reliable change over the three assessments.

Finally, moderation analyses were carried out using the S [66]. The dependent variable was the change in attachment and emotional skills from pre- to post-intervention (T2-T1), and the independent variable was the experimental condition. Demographic variables (gender and age) and relationship variables (relationship duration and relationship satisfaction) were introduced separately as moderating variables. In the case of a significant interaction effect between the independent variable and the moderating variable, the latter is considered to influence the change and must be considered.

### 2.5. Description of EMOVERE: Intervention Program for Couples

EMOVERE program is a weekly, seven-session intervention lasting 14 h. The main objective of EMOVERE is to increase the intrapersonal and interpersonal emotional competences of young couples. The program is developed in groups of 8–10 young couples and has theoretical and practical interactive activities in individual, couple and group formats. Psychoeducation in emotional competences is transversal to the entire program, and the experiential work of the participants is also encouraged. Two psychologists implemented the intervention after receiving specific training. At the end of each session, facilitators offer participants materials and different proposals focused on generalizing what they have learned to their everyday lives. The aim and activities developed in each session are detailed in Table 1.

In the EMOVERE program, we work with the three factors that the vulnerability–stress–adaptation model [52] includes. Regarding the “vulnerability” factor, we work to identify and understand both the participant’s own and the couple’s style of attachment, we increase awareness of the participant’s own communication style and explore how each person has learned to relate to emotions from childhood to the present. As for the “stress” factor, throughout the program, we invite each couple to apply the contents to their own reality, reflecting on concrete examples from their lives, and sharing their concerns with the group. Finally, we encourage the “adaptation” factor by identifying the strengths of each couple and training with the psychological resources learned in the sessions.

As mentioned above, the emotion regulation model based on emotional processing [53] was adapted for couples from an interpersonal perspective, as follows: (1) emotional openness: ability to honestly express to the partner the sensations and emotions detected in one’s own body; (2) emotional attention: ability to dedicate time to pay attention to partner’s emotions, considering verbal and non-verbal communication that indicates changes in their emotional states; (3) emotional acceptance: ability to accept and validate the partner’s emotions, without ignoring or criticizing; (4) emotional labeling: ability to understand the partner’s emotions, and help to break down their emotions to gain clarity; (5) emotional analysis: ability to understand the message behind the partner’s emotions, and to analyze its origin in order to understand its meaning; and (6) emotional modulation: the ability to contain the emotional overflow in conflicts, as well as having resources to soothe the partner and return to calm and security after an intense emotional experience. Throughout the program, participants are guided on a progressive route through the different processes, putting them into practice.

## 3. Results

### 3.1. Effectiveness of the Intervention Program

There appear to be no statistically significant differences in T1 between participants from the experimental and control group in terms of gender (χ2 = 0.05; *p* = 0.831), age (χ2 = 22.20; *p* = 0.177), sexual orientation (χ2 = 3.12; *p* = 0.374) and relationship duration (t = 0.97; *p* = 0.337).

The results of the analysis of multivariate variance (MANOVA) indicate that there are no previous differences between the control group and the experimental group in any of the variables before the intervention (Wilks’ lambda; λ = 0.93; F = 0.49; *p* = 0.907). Thus, it could be stated that both groups are homogeneous before the application of the intervention in their levels of emotional intelligence, emotional inter-regulation with their partner and attachment security (Table 2).

The results of the multivariate covariance analysis (MANCOVA) indicate that there are statistically significant differences between the control group and the experimental group after the intervention (Wilks’ lambda, λ = 0.73; F = 2.15; *p* = 0.029). As for intrapersonal emotional intelligence, participants in the experimental group presented higher levels of emotional repair (F = 5.57; *p* = 0.021). Regarding the interpersonal emotional competences, participants in the experimental group presented higher levels of emotional expression to the partner (F = 12.12; *p* = 0.001), understanding of the partner’s emotions (F = 9.96; *p* = 0.002) and regulation of the partner’s emotions (F = 7.89; *p* = 0.006). Regarding the subjective perception of the partner’s emotional inter-regulation competences, the participants in the experimental group affirmed that their partners showed greater emotional understanding (F = 8.31; *p* = 0.005). Likewise, participants in the experimental group showed lower levels of avoidance of intimacy than those in the control group (F = 10.42; *p* = 0.002). The size of the effect of the differences was low–moderate, ranging from d = 0.10 to d = 0.39. The mean of effect size in T2 considering those variables with significant effects (repair, avoidance of intimacy, expression actor, understanding actor, management actor and understanding partner) was moderate (d = 0.24).

### 3.2. Multiple Hierarchical Regression

In the first step of the multiple hierarchical regression, the results indicated that the initial level influences the level of change (Table 3). In this sense, when there is a higher level in T1, there is less change, while when the basal level is lower, there is more change (β = [−0.21, −0.60]). By controlling the influence of T1, the second step shows the influence of the condition (experimental group vs. control group) on the change. The positive regression coefficient indicates that the higher the condition (1 > 0), the greater the change.

The results indicated that belonging to the experimental group influenced the change from T1 to T2 in certain variables compared to the control group. Specifically, the participants of the experimental group increased the intrapersonal emotional repair (β = 0.23; t = 2.32; *p* ≤ 0.05), the expression of emotions to the partner (β = 0.30; t = 3.12; *p* ≤ 0.01), the understanding of emotions of the partner (β = 0.20; t = 2.13; *p* ≤ 0.05) and the regulation of emotions with the partner (β = 0.24; t = 2.49; *p* ≤ 0.05). They also increased the perception that the partner can understand one’s own emotions (β = 0.20; t = 2.27; *p* ≤ 0.05). Finally, participants in the intervention decreased their avoidance of intimacy (β = -0.25; t = −2.54; *p* ≤ 0.05).

### 3.3. Reliable Change Index (RCI)

The results show that the experimental group consistently presents higher percentages of real change than the control group (Table 4). In emotional intelligence, there was a real change for three people (6.8%) in attention, five people (11.4%) in clarity and seven people (13.6%) in repair in the experimental group, while in the control group, there was real change in one person (2.3%), two people (4.5%) and four people (9.1%), respectively. In attachment, there was a real change in six people (13.6%) in anxiety and in eight people (18.2%) in avoidance in the experimental group, while in the control group, there was no real change in any person in avoidance and in two people in anxiety (4.5%).

Regarding the emotional inter-regulation of the actor (who responded to the questionnaire), in the experimental group, five people (11.4%) improved in expression, six (13.6%) in understanding and seven (15.9%) in management, while in the control group, no person improved in expression and management, and one person (2.3%) in understanding. Regarding the emotional inter-regulation of the partner (the perception the actor has about their partner), in the experimental group, six people (13.6%) claimed their partners improved in expression, 10 people (22.7%) in understanding and nine (20.5%) in management, while in the control group, real change occurred in two people (4.5%) in expression and one (2.3%) in management, and none of them improved in understanding. The percentage of people who showed a reliable change was significantly higher in the experimental group than in the control group in the following variables: avoidance of intimacy (χ2 = 9.39; *p* ≤ 0.01), emotional expression of the actor (χ2 = 7.61; *p* ≤ 0.05) and emotional management of the actor (χ2 = 7.62; *p* ≤ 0.05), as well as understanding of the partner (χ2 = 11.98; *p* ≤ 0.01) and management of the partner (χ2 = 9.72; *p* ≤ 0.01).

In summary, if we take an average of the rates of reliable change of the experimental group and the control group in the eleven variables studied, we can say that 14.87% of the experimental group has shown a reliable change, compared to 1.18% in the control group.

### 3.4. Moderating Variables of the Effectiveness of the Intervention Program

Moderation analyses were performed (Table 5), showing that there is an interaction effect of age (b = 0.07; t = −2.57; *p* ≤ 0.05; 95% CI = (−0.15, −0.02)) and relationship satisfaction in improving repair in the experimental group (b = 0.04; t = 2.02; *p* ≤ 0.05; 95% CI = (0.01, 1.26)). Likewise, an interaction effect of the duration of the relationship in the change of avoidance is also observed (b = 0.04; t = 2.06; *p* ≤ 0.05; 95% CI = (0.00, 0.02)).

## 4. Discussion

The quality of a sentimental relationship has a strong influence on health and well-being [6,19]. There is a social need to educate people on how to establish healthy and violence-free relationships from youth, when the first stable couple relationships begin to be established [7]. One way of strengthening quality bonds is the development of intrapersonal and interpersonal emotional competences [10]. Therefore, the objective of this study was to assess the effectiveness of EMOVERE, a program to increase emotional competences of young couples. Our intervention seems to be effective in increasing both emotional self-regulation and emotional inter-regulation abilities with a partner, as well as promoting intimacy within the couple.

### 4.1. The Effectiveness of the Program

The results obtained suggest that our program is effective in improving intrapersonal and interpersonal emotion regulation with a partner. We can say that the first proposed hypothesis is fulfilled, although not completely. The program manages to effectively increase the capacity to regulate one’s emotions. This means that participants increased their ability to influence the intensity of their emotions and the way they are experienced. In addition, they increased their ability to adequately express emotions to their partner, attend and understand their partner’s emotions, as well as validate and soothe their partner’s emotions. Additionally, after completing the program, participants reported that they perceived their partners as having greater ability to understand them emotionally, which is likely to increase the perception of satisfaction [8]. Thus, although some intrapersonal emotional abilities have not been explicitly increased after the program (such as attention to emotions or emotional understanding), according to the emotional processing model [53], we could deduce that these skills are implicit in emotion regulation, the most complex ability, which is indeed strengthened by the program [13].

Previous studies suggest that people with insecure attachment, both anxious and avoidant, have greater difficulties in understanding and regulating their own and their partners’ emotional states than people with a secure attachment style [18]. The second hypothesis was that the program would serve to increase couples’ secure attachment, reducing attachment anxiety and avoidance of intimacy. This hypothesis is partially fulfilled, demonstrating its effectiveness in reducing avoidance of intimacy. This means that participating together in the sessions increases the perception of intimacy with and trust in the partner and less tendency to isolate oneself or to engage in emotionally deactivating behaviors in the face of conflicts [17]. This may be because the program provides an opportunity to couples to experience exercises with a high level of intimacy, where they can express their emotions and needs in an honest way. As far as attachment anxiety is concerned, the program may need to increase the resources offered to reduce the insecurity and fear of abandonment characteristic of this style [17].

Regarding the size of the average effect of the results in our study, we can say that it is in line with previous studies. The meta-analysis by Simpson, Leonhard and Hawkins [23] found significant mean effect sizes (studies with control group d = 0.36; studies with pre-post d = 0.47), including effects on attitudes and skills. In our study, the mean effect size is d = 0.24. This means that, although there seems to be a trend towards change, it is moderate and will only occur in a smaller proportion of participants. Regarding this proportion, we observed that 14.87% of the experimental group has shown a reliable change, compared to 1.18% in the control group.

The third hypothesis suggested that demographic characteristics as well as the initial levels of relationship satisfaction could moderate the effectiveness of the intervention, which is confirmed by the results. Age and relationship satisfaction appear to be moderating participants’ learning of emotional self-regulation. Participants who learn to self-regulate their emotions most easily are the younger ones and those most satisfied with their relationships. This could be because younger people have greater plasticity for learning [4]. Likewise, a high prior relationship satisfaction facilitates emotional management, probably because the intensity of negative emotions is lower than in those couples where dissatisfaction prevails [20]. The duration of the relationship seems to be moderating the changes in attachment behaviors. Longer-lasting couples have reduced their avoidance of intimacy more than shorter-lasting couples. That is, it seems that those couples who frequently used deactivation emotion regulation strategies in the face of emotions have managed to become more intimate thanks to the program [16]. It is also important to note that gender is not a moderating variable, so men and women seem to benefit equally from our program.

### 4.2. Strengths, Limitations and Future Considerations

Considering the criticism that previous prevention programs for couples have received, the EMOVERE program takes up and implements the various suggestions for improvement that have been proposed [51]. One of the strengths of the program is the systematization of its design, since all sessions were strictly applied by the same psychologist previously trained in the program [44]. In addition, unlike some previous studies, we have theoretical and methodological soundness, since a pre–post design with a comparison group was carried out. Since some couples are discouraged from participating in interventions by barriers such as cost or the amount of time required [52], so the EMOVERE program was open and free. In addition, time availability was adjusted to the interested couples’ own demands, and the duration was short. Considering that the most suitable time for interventions of this type is between 9 and 20 h, our program was 14 h [44]. Considering that there is often a high dropout rate from programs and reduced effectiveness when participants miss certain sessions, participant adherence was encouraged, achieving a retention rate of 84.62% [35].

However, the limitations of the study cannot be ignored. One of the main limitations is the small sample size, which should be expanded in future research, as well as diversified (e.g., to include more couples of non-normative sexual orientations, from different geographical locations in Spain and other sociodemographic characteristics, such as economic level or cultural background). Thus, we can see that our results can be generalized to a young Spanish population with different characteristics.

Another of the main limitations of our study is the absence of a medium- or long-term follow-up assessment since we only have a post-short-term measure. Therefore, we cannot bring clarity to the contradiction of results existing in the previous literature on the doubtful medium- and long-term effects of relationship education programs [67]. It would be advisable to carry out another phase of this study in which the evolution of the variables studied in both the control and experimental groups are evaluated.

On the other hand, it is also remarkable that this is non-blinded research, since the psychologist who carried out the program sessions knew the objective of the research, which could have influenced the results by experimenter effects, such as generating social desirability in the participants. For future applications of the program, it would be of interest that it be applied by different trained psychologists who are not involved in the research itself.

Finally, we can observe that the effect sizes in our research are small to moderate. This is a frequent result in psychoeducation programs, as it is often difficult to observe large changes in psychological variables [23]. Therefore, although the results are encouraging, it is necessary to be careful with their interpretation. Moreover, there is an evident need to keep on improving the quality of relationship education programs to maximize their effects and benefits.

Despite the limitations, we believe that the program has interesting practical implications for other researchers, educators and psychologists. The EMOVERE program could be applied in the university context, as an activity outside the academic curriculum to reinforce the well-being of university youth. It could also be useful to be applied in high schools, extending the study of its outcome to the adolescent population. Clinical psychologists in the private sphere could make use of the program to prevent the development of couple problems and promote health. In general, other organizations and associations in both the public and private sectors interested in positive youth development could use our research to work with young populations. To carry out these proposals, the authors are available for other researchers and professionals who want to request the complete program to continue with the assessment of its effectiveness in diverse and more extensive samples.

To sum up, this work aims to awaken the interest of psychologists and researchers in the emotional education of young couples [16]. Effective intervention programs appear to be useful for young people to learn to express, understand and regulate their emotions as a couple, and, as a consequence, establish higher-quality relationships [50]. This may be an appropriate way to enhance the general health and well-being of this population, and, at the same time, prevent social problems such as partner violence [7].

## 5. Conclusions

In conclusion, we can highlight the main results obtained after the application of our EMOVERE program for emotional education in young couples. Regarding intrapersonal emotional competences, the results suggest that our program effectively increases emotional self-regulation. Within interpersonal emotional competences, the program seems to improve emotional expression to the partner, as well as the partner’s understanding and management of the partner’s emotions. Additionally, participating in the program increases the perception of the partner’s emotional understanding. Finally, it seems to reduce couples’ avoidance of intimacy. Age, relationship duration and relationship satisfaction moderate the effectiveness of the program. These findings could be of interest to complement existing literature on emotional education and effective intervention for couples.

## Figures and Tables

**Table 1:** Description of EMOVERE program sessions.

Session	Objectives	Activities
1	Presentation and contextualization of the program	- Presentation to the group of our identity as a couple using metaphors. ^PA, GA^ - What is “being a couple” and what are the pillars of a relationship. ^IA, PA, GA^ - “Who we are”: a dynamic exercise in group cohesion. ^GA^ - Getting in touch with the emotion regulation model. ^GA^ - Tip for the week: Stan Tatkin video display and exercise to encourage intimacy. ^PA^
2	Development of openness and emotional attention	- Focusing of body sensations and emotions. ^IA^ - Self-analysis: facilities and difficulties to perceive my emotions. ^IA, PA, GA^ - Guided meditation for couples: “How can you open my chest of emotions”. ^PA^ - Guidelines and practice of active listening. ^GA^ - Emotional observation of my partner (verbal and non-verbal communication). ^PA^ - Tip for the week: recommended reading and self-registering. ^IA^
3	Practice of acceptance and labeling of emotions	- A tour of the basic emotions and my relationship with them during my life. ^PA^ - Universe of emotions: learning complex emotional vocabulary. ^GA^ - Game of “emotional taboo” for the integration of emotional vocabulary. ^PA^ - Guidelines and practice of emotional validation of my partner. ^PA, GA^ - Artistic activity: modeling in plasticine my present emotion. ^IA, PA^
4	Awareness raising of attachment styles	- “Looking for your hug”: experiential exercise of each attachment style. ^PA, GA^ - Learning the main characteristics of attachment styles. ^GA^ - Identification of each partner’s attachment style. ^IA, PA^ - Use of body expression to represent feared and desired scenes in the couple. ^PA, GA^ - Expression of intimate needs to the partner by the “position of the lovers”. ^PA^ - Tip for the week: “write my life story in chapters”. ^IA^
5	Practice of emotional analysis and management	- Reading and analysis of the story “The emotional messenger”. ^GA^ - Dual meaning messages through the visualization of movie scenes. ^GA^ - Training in emotional analysis through flowcharting. ^IA, PA^ - Exploring and identifying patterns of “dirty arguing” and “clean arguing”. ^PA, GA^ - Guidelines and practice of conflict management skills with real situations. ^PA, GA^
6	Development of emotion regulation	- Exploration of the 5 languages of love as ways to regulate ourselves as a couple. ^IA, PA, GA^ - Agreement of a “reassuring symbol” to end the escalation of aggression. ^PA, GA^ - “Breaking the cardboard heart”: recognizing emotional damage. ^PA^ - “Pasting the cardboard heart”: showing willingness to repair emotional damage. ^PA^ - Tip for the week: write a letter of thanks to my partner. ^IA^
7	Consolidation of learning and closure	- Review of learning following the model of emotion regulation. ^GA^ - “We are a team”: exploration of the strengths of our relationship. ^PA, GA^ - “Instruction manual of this relationship”: our preferred ways of regulating. ^PA, GA^ - Our commitments from now on to increase our well-being. ^PA, GA^ - Intimate reading of the letter of thanks to the partner. ^PA^ - Sharing of experiences and farewell to the group. ^GA^

Note. ^IA^: Individual activity; ^PA^: Partner activity; ^GA^: Group activity.

**Table 2:** Impact of the intervention program comparing the experimental group and the control group in T1 and T2 on emotional intelligence, attachment and inter-regulation.

Variable	Time	Experimental Group	Control Group	Cohen’s *d* [95% CI]	ANOVA		ANCOVA	
		*M (SD)*	*M (SD)*		*F*	*p*	*F*	*p*
AT	T1	2.93 (0.92)	3.14 (0.86)	−0.24 [−0.42, −0.06]	1.20	0.276	0.60	0.441
	T2	2.87 (0.94)	3.18 (0.87)	−0.35 [−0.53, −0.16]				
CL	T1	3.48 (0.72)	3.55 (0.81)	−0.09 [−0.25, 0.07]	0.20	0.653	1.58	0.213
	T2	3.62 (0.71)	3.57 (0.93)	0.06 [−0.11, 0.23]				
RE	T1	3.34 (0.77)	3.31 (0.79)	0.04 [−0.12, 0.20]	0.02	0.878	5.57	0.021
	T2	3.57 (0.78)	3.27 (0.78)	0.39 [0.23, 0.55]				
ANX	T1	3.45 (1.00)	3.42 (1.12)	0.03 [−0.19, 0.25]	0.02	0.882	0.10	0.756
	T2	3.33 (1.13)	3.35 (1.11)	−0.02 [−0.25, 0.21]				
AV	T1	2.16 (0.69)	2.04 (0.60)	0.19 [0.05, 0.32]	0.86	0.357	10.42	0.002
	T2	1.96 (0.70)	2.15 (0.72)	−0.27 [−0.42, −0.12]				
EX-A	T1	4.07 (0.81)	4.31 (0.62)	−0.34 [−0.49, −0.19]	2.50	0.118	12.12	0.001
	T2	4.23 (0.73)	4.11 (0.67)	0.17 [0.03, 0.32]				
UN-A	T1	4.17 (0.59)	4.26 (0.54)	−0.16 [−0.28, −0.04]	0.57	0.453	9.96	0.002
	T2	4.34 (0.57)	4.21 (0.53)	0.24 [0.13, 0.35]				
MA-A	T1	3.87 (0.70)	4.12 (0.59)	−0.39 [−0.52, −0.26]	3.30	0.073	7.89	0.006
	T2	4.20 (0.67)	4.14 (0.57)	0.10 [−0.03, 0.23]				
EX-P	T1	3.95 (0.79)	4.06 (0.78)	−0.14 [−0.30, 0.02]	0.41	0.525	1.87	0.176
	T2	4.15 (0.74)	4.05 (0.60)	0.15 [0.01, 0.29]				
UN-P	T1	3.98 (0.81)	4.11 (0.70)	−0.17 [−0.33, −0.02]	0.69	0.409	8.31	0.005
	T2	4.20 (0.62)	4.04 (0.66)	0.25 [0.12, 0.39]				
MA-P	T1	3.84 (0.86)	4.05 (0.66)	−0.28 [−0.44, −0.12]	1.61	0.209	1.97	0.165
	T2	4.12 (0.71)	4.14 (0.61)	−0.03 [−0.17, 0.12]				

Note. AT = attention; CL = clarity; RE = repair; ANX = attachment anxiety; AV = avoidance of intimacy; EX-A = expression actor; UN-A = understanding actor; MA-A = management actor; EX-P = expression partner; UN-P = understanding partner; MA-P = management partner; *M* = mean; *SD* = standard deviation; Cohen’s *d* = effect size; CI = confidence interval; *F* = F ratio; *p* = probability; T1 = pre-intervention; T2 = post-intervention.

**Table 3:** Influence of T1 and condition (experimental group vs. control group) on change from T1 to T2 in emotional intelligence, attachment and inter-regulation.

Outcome Variables	Regression: Model 1				Regression: Model 2				
	*ΔR^2^*	*ΔF*	β	*t*	*ΔR^2^*	*ΔF*	β	*t*	*DW*
AT	0.07	6.51 *	−0.27	−2.55 *	0.02	1.38	−0.12	−1.18	2.35
CL	0.06	5.74 *	−0.25	−2.40 *	0.01	0.92	0.10	0.96	1.52
RE	0.15	14.68 ***	−0.38	−3.83 ***	0.05	5.38 *	0.23	2.32 *	1.89
ANX	0.04	3.95 *	−0.21	−1.99 *	0.00	0.11	−0.04	−0.33	2.36
AV	0.10	9.18 **	−0.31	−3.02 **	0.06	6.47 *	−0.25	−2.54 *	1.85
EX-A	0.16	16.47 ***	−0.40	−4.06 ***	0.09	9.72 **	0.30	3.12 **	1.83
UN-A	0.20	21.67 ***	−0.45	−4.66 ***	0.04	4.53 *	0.20	2.13 *	1.98
MA-A	0.19	19.90 ***	−0.43	−4.46 ***	0.06	6.21 *	0.24	2.49 *	1.66
EX-P	0.36	47.47 ***	−0.60	−6.89 ***	0.01	1.53	0.12	1.24	1.86
UN-P	0.33	42.81 ***	−0.58	−6.54 ***	0.04	5.17 *	0.20	2.27 *	1.88
MA-P	0.31	38.01 ***	−0.55	−6.16 ***	0.01	0.95	0.09	0.97	2.02

* *p* ≤ 0.05. ** *p* ≤ 0.01. *** *p* ≤ 0.001. Note. AT = attention; CL = clarity; RE = repair; ANX = attachment anxiety; AV = avoidance of intimacy; EX-A = expression actor; UN-A = understanding actor; MA-A = management actor; EX-P = expression partner; UN-P = understanding partner; MA-P = management partner; T1 = pre-intervention; T2 = post-intervention; ΔR^2^ = change in R^2^; ΔF = change in F; β = regression coefficient; t = value of *t*-test statistic; DW = Durbin–Watson test. Model 1: predictor = pre-intervention score; Model 2: predictor = experimental condition, controlled for pre-intervention score; outcome variables: changes in scores pre- to post-intervention were used for each for the regression analyses. Standardized beta values are reported.

**Table 4:** Differences between the experimental group and the control group in actual change in emotional intelligence, attachment and inter-regulation.

Variable	*χ^2^*	Experimental Group			Control Group		
		RC *n* (*%*)	WRC *n* (*%*)	NRC *n* (*%*)	RC *n* (*%*)	WRC *n* (*%*)	NRC *n* (*%*)
AT	1.05	3 (6.8)	39 (88.6)	2 (4.5)	1 (2.3)	41 (93.2)	2 (4.5)
CL	2.61	5 (11.4)	36 (81.8)	3 (6.8)	2 (4.5)	41 (93.2)	1 (2.3)
RE	2.10	7 (15.9)	35 (79.5)	2 (4.5)	4 (9.1)	35 (79.5)	4 (11.4)
ANX	4.46	6 (13.6)	36 (81.8)	2 (4.5)	2 (4.5)	42 (95.5)	0 (0.0)
AV	9.39 **	8 (18.2)	34 (77.3)	2 (4.5)	0 (0.0)	43 (97.7)	1 (2.3)
EX-A	7.61 *	5 (11.4)	37 (84.1)	2 (4.5)	0 (0.0)	44 (100)	0 (0.0)
UN-A	4.69	6 (13.6)	37 (84.1)	1 (2.3)	1 (2.3)	40 (90.9)	3 (6.8)
MA-A	7.62 *	7 (15.9)	36 (81.8)	1 (2.3)	0 (0.0)	43 (97.7)	1 (2.3)
EX-P	2.27	6 (13.6)	35 (79.5)	3 (6.8)	2 (4.5)	38 (86.4)	4 (9.1)
UN-P	11.98 **	10 (22.7)	32 (72.7)	2 (4.5)	0 (0.0)	39 (88.6)	5 (11.4)
MA-P	9.72 **	9 (20.5)	33 (75.0)	2 (4.5)	1 (2.3)	43 (97.7)	0 (0.0)

* *p* ≤ 0.05. ** *p* ≤ 0.01. Note. AT = attention; CL = clarity; RE = repair; ANX = attachment anxiety; AV = avoidance of intimacy; EX-A = expression actor; UN-A = understanding actor; MA-A = management actor; EX-P = expression partner; UN-P = understanding partner; MA-P = management partner; *χ^2^* = value of chi-square test statistic; *n =* number of cases; *% =* percentage. RC = reliable change; WRC = without reliable change; NRC = negative reliable change.

**Table 5:** Moderator analyses of emotional intelligence and well-being pre- to post-intervention (moderator x group interaction effects).

Change	Statistics	Gender	Age	Relationship Duration	Satisfaction with the Relationship
∆ AT	b	0.00	0.00	0.02	0.00
	t	−0.04	0.05	1.22	0.37
	95% CI	−0.49, 0.47	−0.06, 0.06	−0.00, 0.01	−0.33, 0.35
∆ CL	b	0.00	0.04	0.01	0.03
	t	0.57	−1.86	−0.68	1.57
	95% CI	−0.34, 0.60	−0.11, 0.00	−0.01, 0.01	−0.12, 1.01
∆ RE	b	0.01	0.07	0.03	0.04
	t	−1.05	−2.57 *	−1.79	2.02 *
	95% CI				
∆ ANX	b	0.00	0.04	0.00	0.01
	t	−0.12	−1.80	0.23	−0.73
	95% CI	−0.59, 0.52	−0.14, 0.01	−0.01, 0.01	−0.92, 0.43
∆ AV	b	0.02	0.01	0.04	0.00
	t	−1.41	0.70	2.06 *	−0.68
	95% CI	−0.78, 0.13	−0.04, 0.08	0.00, 0.02	−0.75, 0.37
∆ EX-A	b	0.02	0.02	0.01	0.00
	t	1.53	−1.33	−1.11	−0.25
	95% CI	−0.09, 0.71	−0.09, 0.02	−0.01, 0.00	−0.56, 0.43
∆ UN-A	b	0.02	0.00	0.03	0.00
	t	1.28	0.02	1.73	1.73
	95% CI	−0.14, 0.65	−0.05, 0.05	−0.01, 0.01	−0.61, 0.33
∆ MA-A	b	0.02	0.01	0.01	0.01
	t	−1.21	0.77	0.74	−0.95
	95% CI	−0.63, 0.15	−0.03, 0.07	−0.00, 0.01	−0.72, 0.25
∆ EX-P	b	0.00	0.00	0.00	0.01
	t	0.34	0.10	−0.19	0.87
	95% CI	−0.50, 0.71	−0.08, 0.08	−0.01, 0.01	−0.41, 1.06
∆ UN-P	b	0.00	0.01	0.00	0.00
	t	−0.12	−0.22	−0.47	0.63
	95% CI	−0.52, 0.46	−0.07, 0.06	−0.01, 0.01	−0.42, 0.81
∆ MA-P	b	0.00	0.01	0.00	0.00
	t	0.15	−0.81	0.35	0.15
	95% CI	−0.45, 0.53	−0.09, 0.04	−0.01, 0.01	−0.55, 0.64

* *p* ≤ 0.05. Note. AT = attention; CL = clarity; RE = repair; ANX = attachment anxiety; AV = avoidance of intimacy; EX-A = expression actor; UN-A = understanding actor; MA-A = management actor; EX-P = expression partner; UN-P = understanding partner; MA-P = management partner; ∆ = change; CI = confidence interval; b refers to the unique contribution of the interaction term (moderator x group) in the prediction of the pre- to post-intervention change score after controlling for the separate effects of group and moderator; t = value of *t*-test statistic.

## Data Availability

Data available on request due to restrictions privacy.
